# An observational study on the impact of overcrowding towards door-to-antibiotic time among sepsis patients presented to emergency department of a tertiary academic hospital

**DOI:** 10.1186/s12873-024-00973-4

**Published:** 2024-04-12

**Authors:** Evelyn Yi Wen Chau, Afliza Abu Bakar, Aireen Binti Zamhot, Ida Zarina Zaini, Siti Norafida Binti Adanan, Dazlin Masdiana Binti Sabardin

**Affiliations:** 1 Department of Emergency Medicine, Universiti Kebangsaan Malaysia, Kuala Lumpur, Malaysia; 2 Department of Emergency Medicine, Hospital Canselor Tuanku Muhriz UKM, Kuala Lumpur, Malaysia; 3 Department of Emergency Medicine, Hospital Kulim, Kedah, Malaysia

**Keywords:** Door-to-antibiotic time, ED overcrowding, In-hospital mortality, Length of hospital stay, Sepsis

## Abstract

**Background:**

The latest Surviving Sepsis Campaign 2021 recommends early antibiotics administration. However, Emergency Department (ED) overcrowding can delay sepsis management. This study aimed to determine the effect of ED overcrowding towards the management and outcome of sepsis patients presented to ED.

**Methods:**

This was an observational study conducted among sepsis patients presented to ED of a tertiary university hospital from 18th January 2021 until 28th February 2021. ED overcrowding status was determined using the National Emergency Department Overcrowding Score (NEDOCS) scoring system. Sepsis patients were identified using Sequential Organ Failure Assessment (SOFA) scores and their door-to-antibiotic time (DTA) were recorded. Patient outcomes were hospital length of stay (LOS) and in-hospital mortality. Statistical analysis was done using Statistical Package for Social Sciences (SPSS) version 26. P-value of less than 0.05 for a two-sided test was considered statistically significant.

**Results:**

Total of 170 patients were recruited. Among them, 33 patients presented with septic shock and only 15% (*n* = 5) received antibiotics within one hour. Of 137 sepsis patients without shock, 58.4% (*n* = 80) received antibiotics within three hours. We found no significant association between ED overcrowding with DTA time (*p* = 0.989) and LOS (*p* = 0.403). However, in-hospital mortality increased two times during overcrowded ED (95% CI 1–4; *p* = 0.041).

**Conclusion:**

ED overcrowding has no significant impact on DTA and LOS which are crucial indicators of sepsis care quality but it increases overall mortality outcome. Further research is needed to explore other factors such as lack of resources, delay in initiating fluid resuscitation or vasopressor so as to improve sepsis patient care during ED overcrowding.

## Background

Emergency Department (ED) as the first patient encounter, plays a crucial role in the initial management of sepsis patients. To increase the rate of sepsis survival, emergency physicians aimed for early sepsis recognition, early fluid resuscitation, early appropriate antibiotics and source control [1]. The latest Surviving Sepsis Campaign guideline 2021 recommended antibiotic time within 1 h for patients with possible septic shock or high likelihood for sepsis. In cases where sepsis is possible but without hypotension or shock, rapid assessment of an etiology should be determined within 3 h. If concern for infection persists, antibiotics should be given within 3 h of sepsis recognition [2].

However, most EDs had failed to achieve the targeted time for antibiotics initiation. Studies show that ED overcrowding delays antibiotics initiation in sepsis patients with an increase of DTA time by 4 min for each 10% increase in ED occupancy [3–6].

ED overcrowding is defined as a situation where the demand for ED services exceeds the ability to provide service [7]. In Malaysia, there are 223 hospitals that provide ED service, but the number of patient visits has been increasing [8]. The escalating demand for ED services had surpassed the rate of ED expansion, hence causing ED overcrowding.

Research on ED overcrowding and DTA time in Malaysia is lacking, despite the extensive publications available from other regions [3, 4, 6, 9–11]. Hence, in this study, we aimed to determine the impact of ED overcrowding towards the DTA time in sepsis patients with LOS and in-hospital mortality as secondary outcomes in a tertiary hospital in Malaysia.

## Method

### Study design & setting

We conducted an observational study, from 18th January 2021 until 28th February 2021 in an 880-bed tertiary academic hospital, Hospital Canselor Tuanku Muhriz (HTCM), Universiti Kebangsaan Malaysia (UKM) in Kuala Lumpur, Malaysia. The annual ED attendance is around 70,000 visits with admission rate of 13% [12]. Sepsis patients account for about 10% of the hospital admissions according to our unpublished internal ED census.

This study was approved by the Medical Research Ethics Committee (MREC) Universiti Kebangsaan Malaysia (JEP-2020-634).

### Selection of participant

The inclusion criteria were all patients above 18 years old who presented to the ED, diagnosed with sepsis and had received antibiotics in ED. The diagnosis of sepsis and septic shock were based on the Third International Consensus Definitions for Sepsis and Septic Shock (Sepsis-3) [1]. Patients with sepsis were defined as patients presented with a source of infection and sustained organ dysfunction. Organ dysfunction is present when two or more criteria in Sequential Organ Failure Assessment (SOFA) were met. SOFA is a scoring system which requires laboratory testing to calculate the dysfunction level in six systems, namely respiratory, cardiovascular, coagulation, liver, renal and neurological systems [1, 13]. Meanwhile, septic shock was defined as patients with persistent hypotension despite adequate fluid resuscitation and requiring vasopressors to maintain MAP ≥ 65mmHg.

All patients presented to ED with probable infection and were prescribed and received antibiotics during this study period were identified and screened for eligibility. Patients who fulfilled the inclusion and exclusion criteria were recruited. A total of three patients who were discharged at their own risk, and four patients who did not receive antibiotics despite antibiotics being prescribed in ED had been excluded from our study.

### Data collection and processing

The study included two parts of data collection, which ran concurrently. The first part involved determination of ED status, using the National ED Overcrowding Study (NEDOCS) scoring system. NEDOCS scoring system measured ED overcrowding using seven variables recorded at one point, which includes total number of patients in ED, number of ED beds, total number of admitted patients in the ED, number of hospital beds, waiting time from triage to ED bed placement for patients placed in ED beds, longest boarding time of patients waiting for admission and number of ventilators in use in ED [14]. Data were collected daily from three shifts at their peak time which were 11am, 6pm, and 11pm daily. Our rationale for this decision was based on the variability in patient acuity and staffing patterns that occur throughout the day, which may impact the delivery of care. By collecting data from three shifts, we aimed to capture a more comprehensive representation of the clinical environment. All data required for NEDOCS scoring were collected from ED bed manager’s census, patient’s case notes and direct observations by the researcher during the designated data collection times. These data were then entered into MedCalc for windows, version 5.2.5 to calculate the NEDOCS scoring of each respective shift.

The severity of ED overcrowding was graded into 6 levels, with level 1 (0–20) being not busy, level 2 (20–60) busy, level 3 (60–100) extremely busy but not overcrowded, level 4 (100–140) overcrowded, level 5 (140–180) severely overcrowded and level 6 (190–200) dangerously crowded. Level 1 to level 3 is grouped as non-overcrowded ED whereas level 4 to level 6 is grouped as overcrowded ED. A pilot study was performed from 30th November 2020 to 6th December 2020 to ensure the feasibility of utilizing NEDOCS in our setting. Throughout the seven days; consisting of 21 shifts; the NEDOCS score ranged from 2 to 6 (median score = 4).

The second part involved data collection of sepsis patients who received antibiotics in ED. During the study period, all patients presented to the ED with probable infection were identified. The treating team provided care to these patients according to standard protocols, and the management instituted were documented in their case notes. Relevant clinical and demographic data like age, gender, race, arrival time, DTA time, and SOFA score were collected during this process. Patients with SOFA score ≥ 2 and who had received antibiotics were recruited in the study. Following the admission of the patients, they were followed up and their LOS and in-hospital mortality were retrieved from the patient’s case note and hospital electronic system database. The NEDOCS score of the ED shifts during which the patients presented were compared and analysed.

The DTA time was measured as the difference between time of patient’s registration to first eligible antibiotic administered. The time was taken as per that recorded in the patient’s drug chart. Hospital LOS were counted from the first day of patient presentation to ED till discharge, where the data were collected from the hospital electronic system databases. In-hospital mortality was defined as patients who died during admission in hospital, in which data were collected from hospital electronic system database and patient’s medical records. The patient’s identifiers were coded and treated with the utmost discretion to maintain the patient’s confidentiality. All treatment and management of patients were under the discretion of the treating physicians as per department protocol.

### Data analysis

The continuous variables were presented as mean ± SD (standard deviation), or median (Interquartile range (IQR)) for duration and clinical parameters, while categorical variables were expressed as frequencies and percentages. To compare sepsis patient presented during ED overcrowded and not overcrowded period, Pearson chi-square test was used for categorical variables like gender, race, ED triage zone, and in-hospital mortality. The Student T test was used for continuous variables with equal distribution like age, diastolic blood pressure, respiratory rate, temperature, GCS, SpO2 and SOFA score. To analyse continuous data variables with unequal distribution, like systolic blood pressure, heart rate, DTA time and length of stay, the Mann-Whitney U test was employed.

Mann-Whitney U test was also used to compare LOS between septic patients and sepsis patient without shock. To analyse in-hospital mortality, Pearson chi-square test was used; while Fisher’s Exact test was used when the data count was below five. Kruskal-Wallis Test was used to compare in-hospital mortality with hour upon hour DTA time among the sepsis patients.

All the tests were two-sided and p-values below 0.05 were regarded as statistically significant. Binary logistic regression is used to estimate the effect of ED overcrowding towards in-hospital mortality. Statistical analysis was carried out using Statistical Package for Social Science (SPSS Statistics 26.0, IBM Corp, Armonk, NY).

## Results

**Fig. 1:**
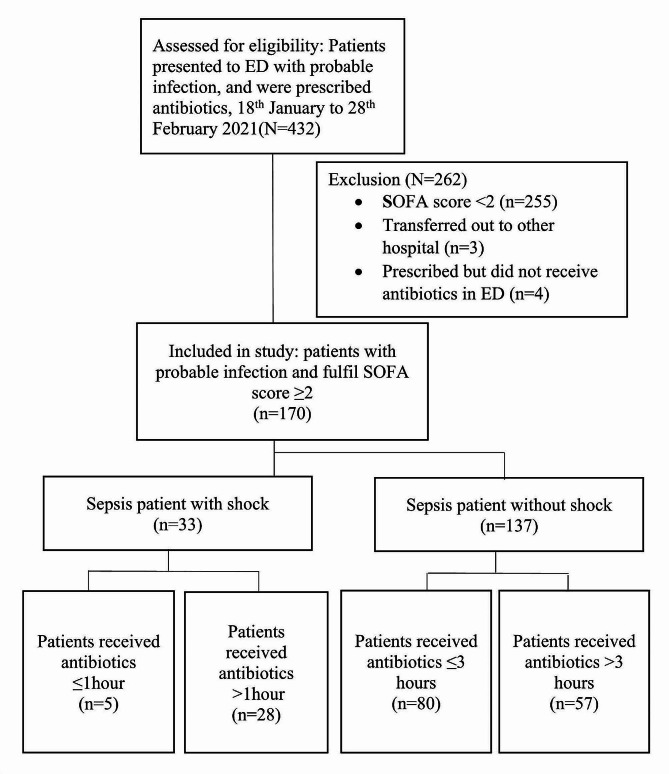
Flow-diagram of sample selection

Data from 126 ED shifts were collected whereby 62 shifts (49.2%) were overcrowded (NEDOCS level 4–6) and 64 shifts (50.8%) were not overcrowded (NEDOCS level 1–3). The most crowded shifts occurred during night shifts (*n* = 65, 51.6%). Weekdays were more crowded compared to weekends (*n* = 109, 87.1%). In these 126 shifts, a total of 432 patients with probable infection had presented to ED, in which only 40% (*N* = 177) fulfilled SOFA criteria ≥ 2. The final recruitment were 170 patients with seven being excluded (Fig. 1).

Generally, the patients who presented with sepsis had a mean SOFA of 3 and were mostly elderly male. A total of 78 patients (45.9%) had presented during overcrowded ED and 92 patients (54.1%) had presented in a not overcrowded ED. There is no statistical difference in the clinical parameters of the patients between each group. The patients’ demographic and clinical parameters were as shown in Table 1.

**Table 1:** Characteristics of patients with sepsis according to ED condition

All patients = 170	ED overcrowded (*N* = 78)		ED not overcrowded (*N* = 92)		p-value
**Gender**					0.426^a^
Female, No. (%)	36	(49.3)	37	(50.7)	
Male, No. (%)	42	(43.3)	55	(56.7)	
**Race**					0.182^a^
Malay, No. (%)	26	(44.1)	33	(55.9)	
Chinese, No. (%)	42	(48.3)	45	(51.7)	
Indian, No. (%)	10	(52.6)	9	(47.4)	
Others, No. (%)	0	(0)	5	(100.0)	
Age, Median in years (IQR)	74	(80.0)	73	(78.0)	0.623^b^
**ED triage zone**					0.362^a^
Red, No. (%)	43	(50.0)	43	(50.0)	
Yellow, No. (%)	32	(43.8)	41	(56.2)	
Green, No. (%)	3	(27.3)	8	(72.7)	
**Clinical parameters**					
SBP, mean in mmHg (SD)	134.1	(45.2)	131.3	(38.3)	0.656^c^
DBP, median in mmHg (IQR)	74.0	(20–145)	72.0	(20–123)	0.919^b^
HR, mean in bpm (SD)	99.7	(25.9)	104.1	(22.1)	0. 231^c^
RR, median in x/min (IQR)	24.0	(7–50)	23.0	(15–45)	0.529^b^
T, median in degree Celsius, (IQR)	37.7	[36–42]	37.4	(35.8–41)	0.151^b^
GCS, median, (IQR)	15	[3–15]	15	[3–15]	0.588^b^
SpO2, median in %, (IQR)	95.0	(50–100)	96.0	(50–100)	0.376^b^
SOFA score, Median (IQR)	3.0	[2–14]	3.0	(0–12)	0.065^b^

*SBP*, systolic blood pressure; *DBP*, diastolic blood pressure; *HR*, heart rate; *RR*, respiratory rate; *T*, temperature; *SD*, standard deviation; *IQR*, interquartile range

*P* < 0.05 is statistically significant

^a^ Pearson chi-square tests

^b^ Mann-Whitney U test

^c^ Student T-test

*Denotes statistical significance of *p* < 0.05

### DTA time and patient’s outcome according to ED overcrowding status

**Fig. 2:**
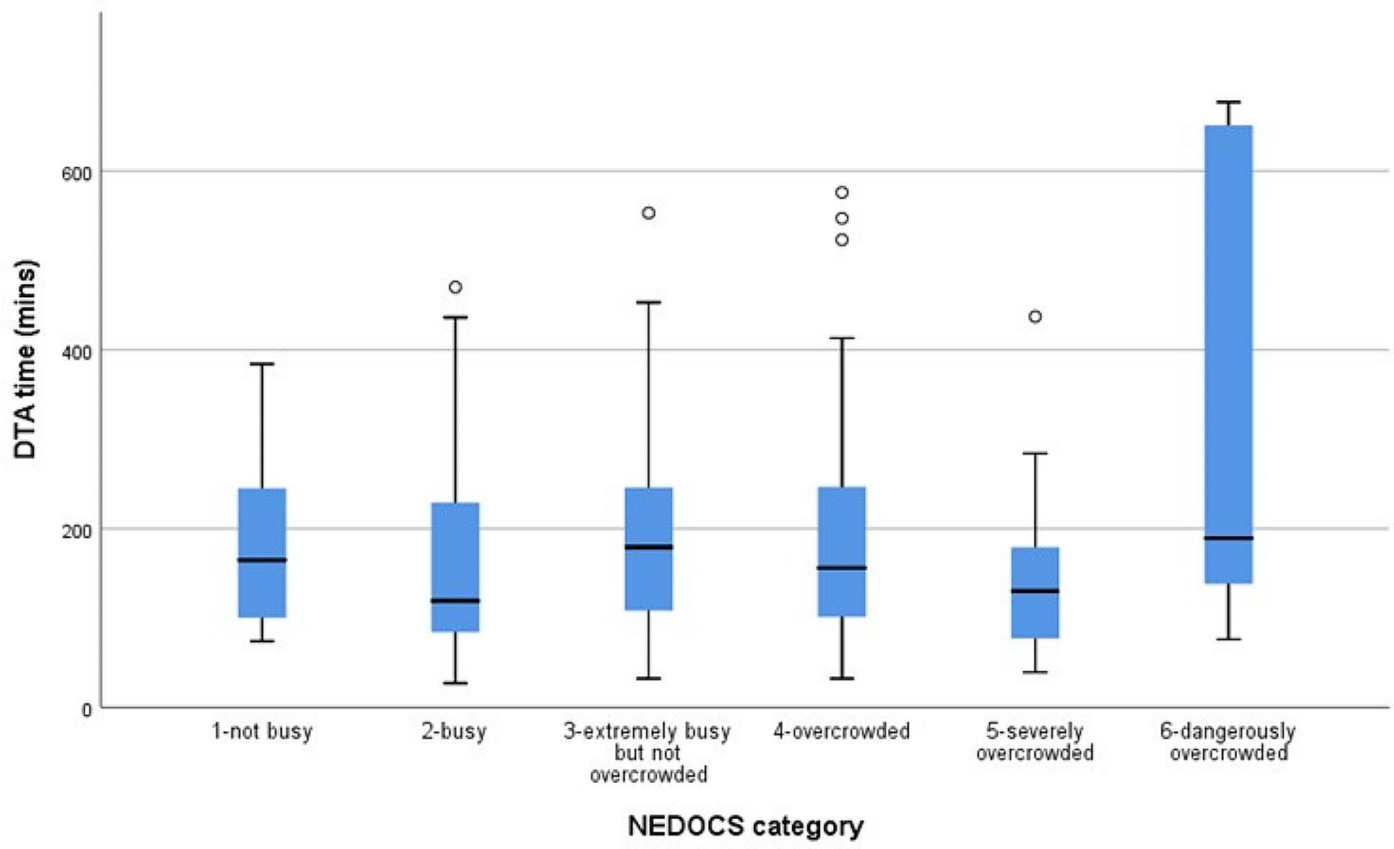
DTA time according to NEDOCS category White dots: outliers

A total of 170 sepsis patients were recruited. Among them, 19.4% (*n* = 33) sepsis patients presented with shock and 80.6% (*n* = 137) patients presented without shock. Only 15.2% (*n* = 5) septic shock patients received antibiotics within 1 h and 58.4% (*n* = 80) sepsis patients without shock received antibiotics within 3 h (Fig. 1). The overall median DTA time was 144 min (IQR 27–677 min). There was no significant difference between DTA in overcrowded ED compared to not overcrowded ED [(median 143 min, IQR 32–677 min) vs. (median 150 min, IQR 27–553 min); p-= 0.989]. There was also no significant difference in DTA time when comparing NEDOCS across categories 1–6 as shown in Fig. 2 (*p* = 0.284). The LOS between these two groups were also not significantly different (*p* = 0.403) as described in Table 2.

**Table 2:** DTA time and patients’ outcome according to ED condition

All patients = 170	ED overcrowded (*N* = 78)	ED not overcrowded (*N* = 92)	p-value
DTA time, median in minutes (IQR)	143 (32–677)	150 (27–553)	0.989^b^
**Patients’ outcome**			
In-hospital mortality, no (%)	27 (34.6)	19 (20.7)	0.041^a*^
LOS, median in days, (IQR)	5 (0–47)	6 (0–25)	0.403^b^

*IQR*, interquartile range

*P* < 0.05 is statistically significant

^a^ Pearson chi-square tests

^b^ Mann-Whitney U test

*Denotes statistical significance of *p* < 0.05

The overall in-hospital mortality was 27% (*n* = 46) with a higher mortality during overcrowded ED compared to non-overcrowded ED (34.6% vs. 20.7%) as described in Table 2. Logistic regression analysis showed that overcrowded ED increased the in-hospital mortality by 2 times (95% CI 1–4; *p* = 0.041). There was no significant association between ED overcrowding and hospital LOS [(median 5 days, IQR 0–47 days) vs. (median 6 days, IQR 0–25 days); *p* = 0.403] as described in Table 2.

Further analysis of the 46 mortalities revealed septic shock group was associated with higher mortality compared to the sepsis without shock group (22.6%, *n* = 31 vs. 45.5%, *n* = 15; *p* = 0.008). There was no significant difference in the hospital LOS between these two groups (*p* = 0.152) as described in Table 3.

**Table 3:** Outcome comparison between sepsis and septic shock patients

	Total sepsis patients (*N* = 170)	Sepsis without shock (*N* = 137)	Septic shock (*N* = 33)	p-value
In-hospital mortality, n (%)	46 (27.0)	31 (22.6)	15 (45.5)	0.008^a*^
LOS, median in days, (IQR)	5 (0–47)	5 (0–47)	5 (0–25)	0.152^b^

LOS, length of stay; IQR, interquartile range

*P* < 0.05 is statistically significant

^a^Pearson chi-square test

^b^Mann-Whitney U test

*Denotes statistical significance of *p* < 0.05

In the septic shock group, there was no significant difference seen between DTA time ≤ 1 h vs. > 1 h and patient’s outcome [in-hospital mortality (*p* = 0.591) and LOS (*p* = 0.673)]. Similarly, the in-hospital mortality (*p* = 0.230) or LOS (*p* = 0.380) in sepsis patient without shock also did not differ statistically between DTA time ≤ 3 h vs. DTA time > 3 h (Table 4).

**Table 4:** Outcome comparison in sepsis patient according to DTA time

Patient’s outcome	Septic shock				Sepsis without shock			
	Total (*N* = 33)	DTA > 1 h	DTA ≤ 1 h	p-value	Total (*N* = 137)	DTA > 3 h	DTA ≤ 3 h	p-value
In-hospital mortality, n (%)	15 (45.5)	13 (39.4)	2 (6.1)	0.591^a^	31 (22.6)	10 (7.3)	21 (15.3)	0.230^c^
LOS, median in days (IQR)	3 (0–25)	2.5 (0–19)	6 (0–25)	0.673^b^	5 (0–47)	2.5(0–19)	6 (0–25)	0.380^b^

LOS, length of stay; IQR, interquartile range

*P* < 0.05 is statistically significant

^a^Fisher’s Exact test

^b^Mann-Whitney U test

^c^Pearson chi-square test

### The impact of hour-to-hour DTA time to in-hospital mortality

**Fig. 3:**
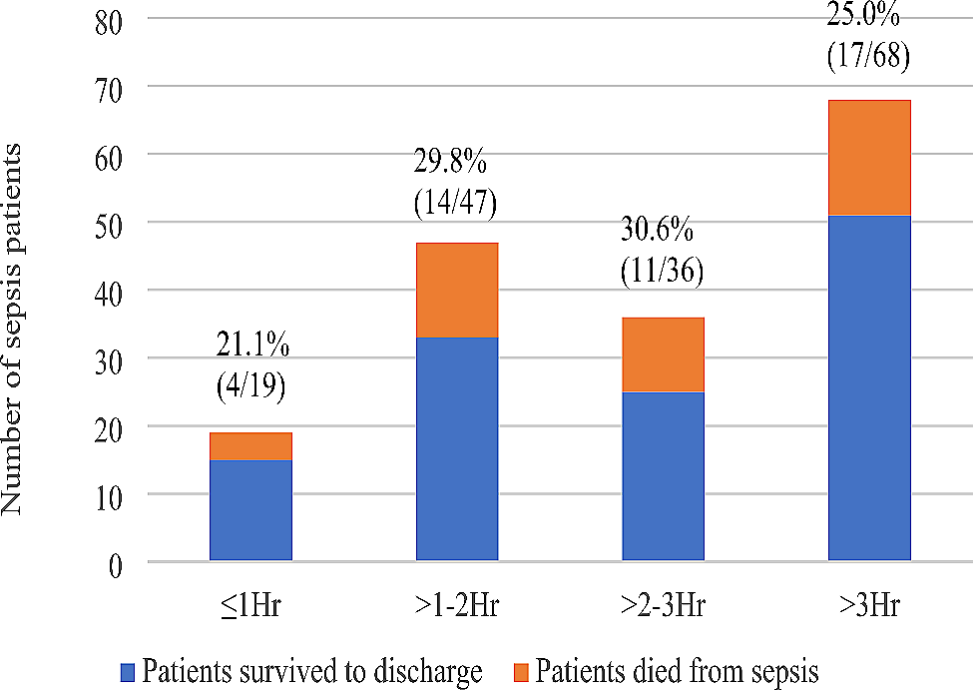
Patient outcome according to DTA time

Figure 3 summarizes the total number of patients that survived or died according to hour upon hour DTA time. The mortality rate of sepsis patients who received antibiotics ≤ 1 h, > 1–2 h, > 2–3 h, >3 h are 21.1%, 29.8%, 30.6% and 25.0% respectively. In general, the mortality rate increased when DTA time was more than one hour, however it was not statistically significant (*p* = 0.827) as shown in Fig. 3.

**Fig. 4:**
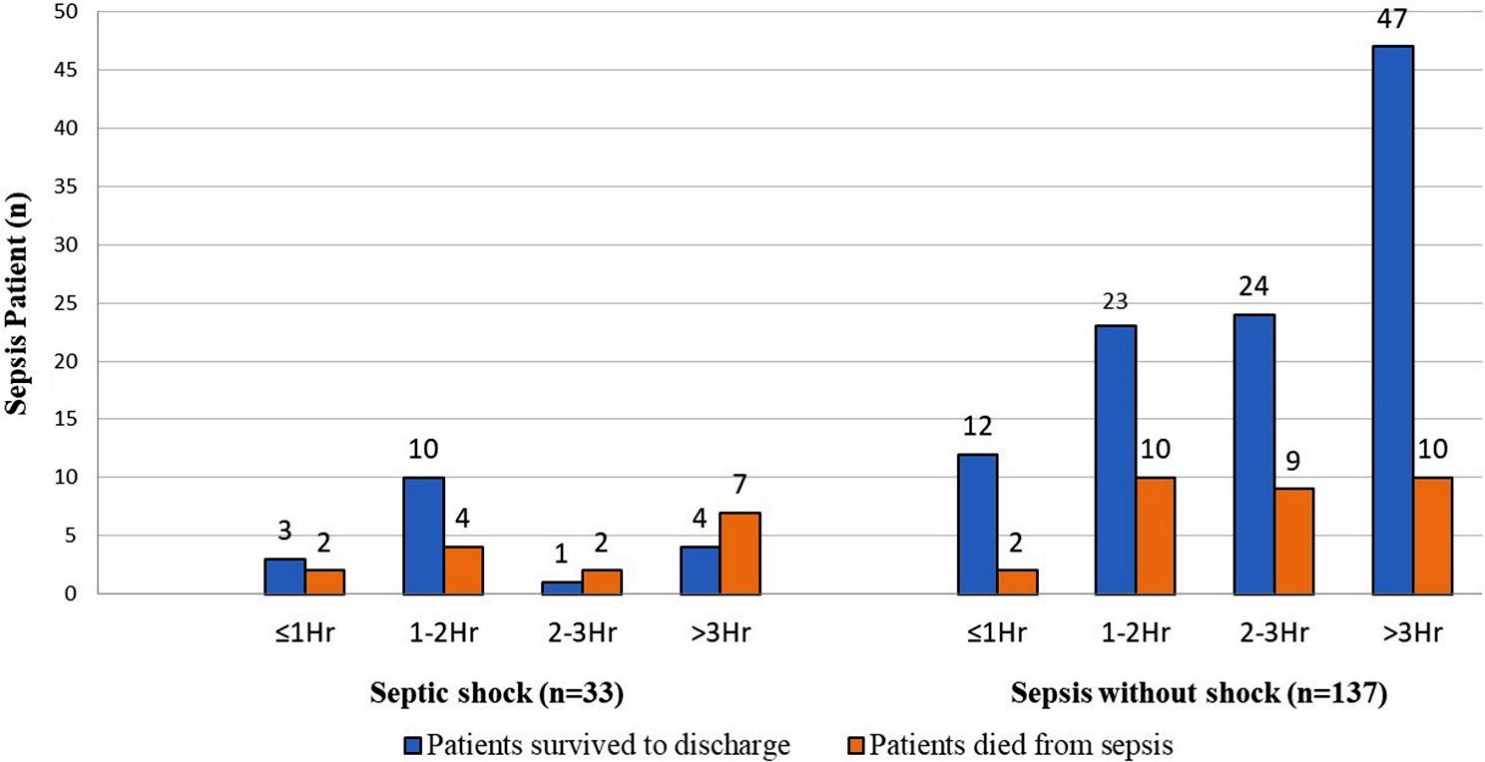
Comparing sepsis groups and their outcomes according to DTA time

The mortality rate of septic shock patients who received antibiotics ≤ 1 h, > 1–2 h, > 2–3 h, >3 h were 40.0%, 28.6%, 66.7% and 63.6% respectively. While the mortality rate for patient with sepsis without shock who received antibiotics ≤ 1 h, > 1–2 h, > 2–3 h, 3 h Were 14.3%, 30.3%, 27.3% and 17.5% respectively (Fig. 4).

## Discussion

ED are designed to deliver time-sensitive interventions for critical illnesses, including sepsis which carries a significant mortality risk [15]. Hence, our study aimed to determine the impact of ED overcrowding towards the DTA time in sepsis patients with LOS and in-hospital mortality as secondary outcomes in a tertiary hospital in Malaysia. In our study, 15% septic shock patients received antibiotics within 1 h and 58.4% sepsis patients without shock received antibiotics within 3 h upon arrival in ED. These timings are notably longer in comparison to other advanced nations such as Korea, Japan and the UK where DTA time within 1 h was achieved in 28.6%, 30.5%, and 48.1% of cases, respectively [16–18]. As a teaching hospital, our ED patients were managed by different levels of doctors; junior doctors, emergency residents and emergency physicians therefore affecting the timings of antibiotics administration [19–21]. Additionally, lack of a standardized sepsis clinical pathway also contributed to the inconsistency in sepsis management, including antibiotics delivery [22–26].

Similar to previous studies done in Thailand and Korea, our study found that the delay in DTA time was not increased further despite ED overcrowding [27, 28]. This might be the result of our ED patient process flow. A systematic approach to all new patients has been enforced in our ED despite the patient occupancy level. In fact, the first doctor to patient contact time is one of the key performance indicators (KPI) of the department. Hence, regardless of the ED condition, all newly arrived sepsis patients will be assessed, investigated, and given the appropriate management such as administration of antibiotics.

The in-hospital mortality rate of 27% found in this study is comparable to other countries such as Korea 28% [29] and China 30% [30]. There was a higher mortality burden in patients with septic shock compared to those without shock and this finding was consistent with previous meta-analysis done [29, 31, 32]. Nonetheless, despite the delay in DTA time, we found no mortality benefit nor reduced hospital LOS in patients who received timely antibiotics. This finding is consistent with meta-analyses done previously, where no significant mortality benefit was found in DTA time within 1 h as compared to 3 h in patients with severe sepsis and septic shock [33, 34]. It is crucial to emphasize that extended delivery times for antibiotics do not necessarily result in poorer outcomes [34–37]. But this does not imply that timely administration of antibiotics is unnecessary. It’s important to approach the implementation of strict time frames with caution. Decisions regarding antibiotic administration should be guided by a comprehensive assessment of each patient’s clinical condition, the susceptibility patterns of the infecting microbes, and the institution’s antimicrobial stewardship policies [3, 38, 39]. Other aspects, such as the duration and volume of fluids administered, the collection of cultures, the types and timing of vasopressors treatments, can also potentially affect sepsis outcomes [37, 39, 40]. However, these factors were not explored in this study.

Although the overcrowded condition in our ED did not result in a significant delay in DTA time, there was two times increase in in-hospital mortality during periods of ED overcrowding. This finding highlights the importance of prompt sepsis management, which includes timely identification of sepsis, hemodynamic support, cultures acquisition, and appropriate antibiotics administration. Continuous monitoring of patients and dynamic reassessment of fluid responsiveness are crucial to prevent complications of fluid overload and reduce risk of mortality [41]. However, during times of ED overcrowding, this critical aspect of sepsis management tends to be overlooked, leading to increased mortality among sepsis patients [3, 42, 43].

### Limitations and recommendations

This study was done in a tertiary teaching hospital with limited sample size. These results were generated from only one hospital and may not be generalizable to all hospitals. The sample size is relatively small especially in the septic shock group with DTA time ≤ 1 h, there were only 2 patients with in-hospital mortality. The small sample size may not accurately represent the outcome for sepsis patients in a larger population. Furthermore, this study may have missed sepsis cases that were not diagnosed during the patient’s stay in ED, resulting in delayed diagnoses that occurred later after the patients were admitted to the ward. We also did not look into other confounding factors that could affect the outcome of sepsis patients, such as the patient comorbidities, severity of sepsis, choice of antibiotic, the duration of antibiotics, and presence of positive cultures. Future studies may be done to explore these factors.

Additionally, our study only demonstrated that there was a delay in DTA, without establishing the exact cause. We recommend future studies to investigate factors that affect DTA time such as pharmacy delays, order processing time between nurses and physicians.

## Conclusions

ED overcrowding is associated with an increase in in-hospital mortality for sepsis patient. However, ED overcrowding did not directly impact LOS and DTA time. Also, the DTA time itself does not affect the in-hospital mortality. This suggest that other factors in the sepsis management pathway may be more critical in determining mortality outcome for sepsis patients during ED overcrowding. Further research is necessary to identify these factors so as to improve survival rate in sepsis patients.

## Data Availability

Data that support the findings of this study have been deposited in the Harvard Dataverse and are available at the following URL: 10.7910/DVN/UNMHX5.

## Abbreviations

ED: Emergency Department
NEDOCS: National Emergency Department Overcrowding Score
SOFA: Sequential Organ Failure Assessment
DTA: door-to-antibiotic
LOS: length of stay
SCC: Surviving Sepsis Campaign
